# Prospective longitudinal study of dynamic depressive symptom trajectories and diabetes onset risk in older adults: a 10-year follow-up of the HRS and ELSA cohorts

**DOI:** 10.3389/fpubh.2025.1656215

**Published:** 2025-10-28

**Authors:** Xiaolin Yu, Yuchen Zhang, Xinran Zhao

**Affiliations:** ^1^Department of Integrated Chinese and Western Medicine, The Affiliated Cancer Hospital of Zhengzhou University and Henan Cancer Hospital, Zhengzhou, China; ^2^School of Traditional Chinese Medicine, Beijing University of Chinese Medicine, Beijing, China

**Keywords:** depressive symptom trajectories, diabetes risk, cognitive-affective symptoms, somatic symptoms, longitudinal cohort study, older adults

## Abstract

**Background:**

The study aimed to examine the longitudinal relationship between depressive symptom trajectories and diabetes onset risk in older adults, with particular attention to sex-specific variations.

**Methods:**

Data were drawn from the Health and Retirement Study (HRS) and the English Longitudinal Study of Ageing (ELSA). Depressive symptoms were measured using CESD-8, and five trajectories were identified: consistently low, decreasing, fluctuating, increasing, and consistently high. Symptoms were further divided into somatic and cognitive-affective domains. Cox proportional hazards models were applied to estimate diabetes onset risk, controlling for demographics, health behaviors, and comorbidities. Analyses stratified by sex were conducted to assess differential effects.

**Results:**

A total of 8,741 participants aged 50 years and older from both cohorts were included. During 10 years of follow-up, increasing (HR = 1.746, 95% CI: 1.195–2.551, *p* = 0.004) and consistently high (HR = 1.376, 95% CI: 1.042–1.818, *p* = 0.024) depressive trajectories were associated with greater diabetes risk compared with the consistently low group. No significant associations were detected for decreasing or fluctuating trajectories. Stronger associations were observed in women, including increasing (HR = 2.007, 95% CI: 1.290–3.121, *p* = 0.002) and consistently high (HR = 1.586, 95% CI: 1.161–2.167, *p* = 0.004) patterns. Similar associations were present across both cognitive-affective and somatic domains.

**Conclusion:**

Persistent or worsening depressive symptoms serve as significant predictors of diabetes onset risk, particularly among women. Both cognitive-affective and somatic domains contribute independently, emphasizing the importance of dynamic mental health surveillance in diabetes prevention.

## 1 Introduction

With population aging, diabetes—one of the most prevalent chronic conditions—has placed a considerable burden on the health and quality of life of middle-aged and older adults (1). According to projections from the International Diabetes Federation (IDF), the global prevalence of diabetes is expected to rise substantially in the coming decades, predominantly affecting these age groups (2). The development of diabetes is shaped by multiple factors, with depression increasingly recognized as a significant psychological contributor linked to its occurrence (3). Large-scale epidemiological studies indicate that depression not only frequently coexists with diabetes but may also act as an independent risk factor for its onset (4, 5). However, the long-term impact of depressive symptom trajectories on diabetes risk in middle-aged and older populations remains unclear.

Most existing research has primarily relied on single-point or baseline assessments of depression to estimate diabetes risk, using static exposure models (6, 7). Such an approach neglects the longitudinal course of depressive symptoms in middle-aged and older adults, thereby disregarding temporal variation and the cumulative impact of emotional distress on metabolic health. Depressive symptoms, however, generally follow dynamic trajectories, including sustained remission, episodic recurrence, or progressive worsening (8). Each trajectory may represent different intensities of psychological stress and physiological adaptation, producing variable influences on diabetes development (9, 10). Although depression has been implicated in metabolic disorders, systematic evaluations of how temporal fluctuations in depressive states shape diabetes onset remain limited (11). Therefore, analyzing depressive symptom trajectories holds considerable public health relevance by clarifying the causal association between depression and diabetes and improving identification of populations at elevated risk.

Depressive symptoms include diverse emotional disturbances, commonly classified into cognitive-affective manifestations (e.g., sadness, hopelessness, social withdrawal) and somatic manifestations (e.g., diminished energy, sleep disturbances, fatigue) (12). These domains capture distinct psychological and physiological facets of depression, each potentially exerting differential effects on health outcomes (13). Although longitudinal studies have provided valuable evidence on the dynamic course of depression, they often treat it as a unidimensional construct and lack detailed investigation of its specific symptom dimensions (14, 15). A dimensional framework and longitudinal tracking of symptom trajectories allow more precise evaluation of the predictive value of particular symptom types, thereby advancing understanding of the bidirectional relationship between mental and metabolic health.

Using long-term data from two large-scale cohorts—the Health and Retirement Study (HRS) and the English Longitudinal Study of Ageing (ELSA)—the present analysis constructed trajectory-based classifications for both overall depressive symptoms and their cognitive-affective and somatic dimensions across multiple survey waves in middle-aged and older adults. Based on these individualized trajectories, subsequent diabetes onset was monitored to systematically assess the associations between depression trajectory types and later diabetes risk. Furthermore, the moderating role of gender within these associations was examined. This investigation clarifies the predictive significance of the longitudinal course of depressive symptoms for metabolic outcomes, providing an empirical basis for early identification and stratified prevention of high-risk individuals in aging populations.

## 2 Methods

### 2.1 Study population

**Figure 1:**
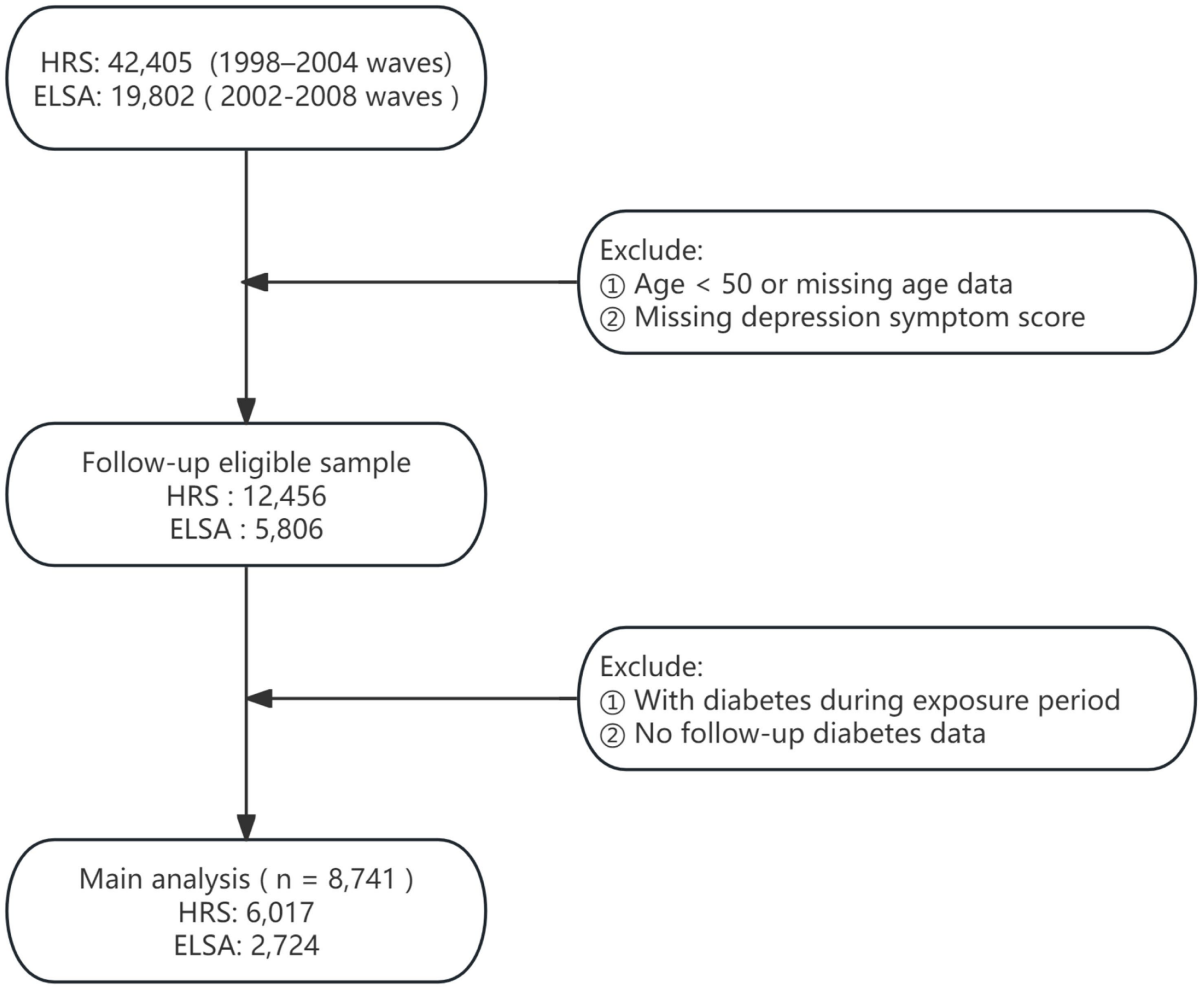
Flowchart of participant inclusion and exclusion.

This study drew upon data from two nationally representative longitudinal surveys: the HRS and ELSA (16, 17). Since 1992, the HRS has conducted biennial assessments of Americans aged 50 and older, collecting extensive information on health, psychological status, economic circumstances, and social determinants. Initiated in 2002, ELSA adopted a design parallel to that of HRS, based on a British national sample, thereby enabling robust cross-national comparisons. Both surveys implement stringent sampling procedures and long-term follow-up, making them highly appropriate for research on aging and health. Ethical approval for HRS was obtained from the University of Michigan’s Institute for Social Research and Survey Research Center, whereas ELSA was approved by the London Multicentre Research Ethics Committee. The detailed study design is presented in Figure 1.

The analytic sample comprised Waves 4–12 of HRS (1998–2014) and Waves 1–9 of ELSA (2002–2018), with Wave 4 of HRS and Wave 1 of ELSA serving as the baseline. Depression trajectories were constructed using HRS Waves 4–7 and ELSA Waves 1–4, allowing classification according to temporal patterns of depressive symptoms. Subsequent incidence of diabetes was assessed using HRS Waves 8–12 and ELSA Waves 5–9.

Exclusion criteria were defined as follows: (1) baseline age younger than 50 years or missing age data; (2) incomplete depression trajectory scores preventing derivation of trajectory indicators; (3) diabetes diagnosed prior to or during the trajectory assessment period; and (4) absence of follow-up information on diabetes onset. Analytical samples that met these conditions were separately established for HRS and ELSA, enabling evaluation of main effects and examination of cross-national consistency.

### 2.2 Primary exposures

The depressive symptoms of respondents were evaluated using a simplified Center for Epidemiologic Studies Depression (CESD)-8 Scale, a simplified version of the Center for Epidemiologic Studies Depression Scale, administered biennially in both the HRS and ELSA cohorts (18). Participants reported the presence of eight symptoms experienced in the past week, with affirmative responses assigned one point each, resulting in a total score ranging from 0 to 8. Higher scores indicated greater severity of depressive symptoms. In line with established criteria, a CESD score ≥3 reliably identified significant depressive symptoms with acceptable sensitivity and specificity (19, 20). Based on this threshold, symptoms were further classified into cognitive-affective and somatic domains (21). The cognitive-affective dimension included three items—“Feeling depressed,” “Feeling lonely,” and “Feeling sad”—which reflected psychological distress and low mood. The somatic dimension consisted of “Everything was an effort,” “Restless sleep,” and “Could not get going,” representing fatigue and functional limitation. Each domain was scored independently on a scale of 0 to 3, with scores ≥2 considered clinically meaningful for the respective symptom category (22, 23).

Based on longitudinal follow-up data, individual-level trajectories were mapped for overall depressive symptoms, cognitive-affective symptoms, and somatic symptoms, reflecting the progression of depressive states across mid- and long-term periods. Trajectory classifications, derived from CESD score patterns across multiple follow-up waves, comprised five categories: (1) consistently low—defined by the absence of high depressive symptoms at all four follow-up waves, with persistently subthreshold CESD scores and no upward trends; (2) consistently high—characterized by elevated depressive symptoms across all waves, with sustained CESD score elevation; (3) increasing—identified as nonelevated depressive symptoms at baseline or the first two waves, followed by persistently elevated symptoms in later waves, suggesting cumulative symptom burden; (4) decreasing—marked by elevated depressive symptoms at baseline or the first two waves, followed by sustained nonelevated symptoms in subsequent waves, indicating symptomatic remission; and (5) fluctuating—including trajectories that did not fit the above categories, typified by irregular variations in CESD scores without consistent directional patterns across the follow-up period.

### 2.3 Outcomes

During follow-up, participants were asked: “Has a doctor ever told you that you have diabetes or high blood sugar?” A diabetes event was defined as any self-reported physician diagnosis of diabetes within a survey wave, and the first such report was recorded as the event occurrence. The outcome time variable was calculated from baseline to either the first diabetes report or the end of the last follow-up, whichever occurred earlier. For participants without a reported event, follow-up time was censored at their final observation.

### 2.4 Covariates

Baseline covariates were included to control for potential confounding, covering demographic and health behavior factors: age, sex, education level (highest degree attained), marital status (married/partnered, separated/divorced/widowed, or single), smoking status (former or never smoker), alcohol consumption history (former or never drinker), and prior heart disease diagnosis. These covariates were measured at baseline or during the exposure period and adjusted in the regression models to improve estimate precision and robustness.

### 2.5 Statistical analysis

All analyses were performed using R (version 4.2.2). Baseline characteristics were summarized by depressive symptom trajectory categories. Continuous variables were presented as mean ± SD, and categorical variables as counts and percentages (*n*, %).

Cox proportional hazards models were applied to assess the relationship between depressive symptom trajectories and diabetes onset risk. The event was defined as the first self-reported diabetes diagnosis during follow-up, with participants lacking such reports censored. Time was measured from the end of the exposure window to either the event or the last follow-up, whichever occurred earlier. Using the consistently low trajectory group as the reference, three models were sequentially specified to adjust for confounders: Model 1 included only the depressive trajectory variable; Model 2 additionally adjusted for demographic characteristics (age, sex, educational attainment, marital status); Model 3 further accounted for health-related behaviors and pre-existing cardiovascular conditions (smoking, alcohol use, and history of heart disease). The proportional hazards assumption was examined using the Schoenfeld residuals test. Missing covariate data were addressed with multiple imputation using the mice package to reduce potential bias.

## 3 Results

### 3.1 Baseline characteristics

A total of 8,741 participants were included and categorized into five trajectory groups according to longitudinal depression scores: consistently low (*n* = 5,566), decreasing (*n* = 142), fluctuating (*n* = 2,575), increasing (*n* = 146), and consistently high (*n* = 312). Baseline demographic and health behavior characteristics for these groups are presented in Table 1.

**Table 1:** Characteristics of participants at baseline.

Variables	Overall	Consistently low	Decreasing	Fluctuating	Increasing	Consistently high
Number (%)	8,741	5,566 (63.7)	142 (1.6)	2,575 (29.5)	146 (1.7)	312 (3.6)
Age (mean (SD))	60.9 (7.2)	61.0 (7.1)	60.7 (7.9)	60.7 (7.4)	60.0 (7.3)	61.1 (7.7)
Sex (%)						
Female	5,477 (62.7)	3,178 (57.1)	104 (73.2)	1838 (71.4)	107 (73.3)	250 (80.1)
Male	3,264 (37.3)	2,388 (42.9)	38 (26.8)	737 (28.6)	39 (26.7)	62 (19.9)
Education (%)						
Below high school	1754 (20.1)	880 (15.8)	51 (35.9)	659 (25.6)	31 (21.2)	133 (42.6)
High school	2,889 (33.1)	1803 (32.4)	49 (34.5)	888 (34.5)	48 (32.9)	101 (32.4)
College or above	3,863 (44.2)	2,744 (49.3)	36 (25.4)	954 (37.0)	64 (43.8)	65 (20.8)
Other	235 (2.7)	139 (2.5)	6 (4.2)	74 (2.9)	3 (2.1)	13 (4.2)
Marital status (%)						
Married or partnered	6,518 (75.5)	4,387 (79.8)	84 (59.6)	1763 (69.4)	112 (78.3)	172 (55.8)
Never married	274 (3.2)	163 (3.0)	7 (5.0)	84 (3.3)	6 (4.2)	14 (4.5)
Separated/divorced/Widowed	1838 (21.3)	947 (17.2)	50 (35.5)	694 (27.3)	25 (17.5)	122 (39.6)
Smoking status (%)						
Ever smokers	4,829 (55.5)	3,026 (54.6)	84 (59.2)	1,458 (57.0)	78 (53.8)	183 (58.7)
Never smokers	3,872 (44.5)	2,516 (45.4)	58 (40.8)	1,102 (43.0)	67 (46.2)	129 (41.3)
Drinking status (%)						
Ever drinkers	5,950 (68.1)	4,007 (72.0)	90 (63.4)	1,620 (62.9)	85 (58.2)	148 (47.4)
Never drinkers	2,790 (31.9)	1,559 (28.0)	52 (36.6)	954 (37.1)	61 (41.8)	164 (52.6)
Heart problems (%)						
No	7,948 (90.9)	5,098 (91.6)	125 (88.0)	2,322 (90.2)	134 (91.8)	269 (86.2)
Yes	793 (9.1)	468 (8.4)	17 (12.0)	253 (9.8)	12 (8.2)	43 (13.8)

The mean age across all groups was approximately 61 years. The proportion of women was highest in the consistently high group (80.1%) and lowest in the consistently low group (57.1%) (*p* < 0.001). Educational attainment followed a similar pattern, with a greater percentage of participants lacking a high school diploma in the consistently high group (42.6%) compared with the consistently low group (15.8%) (*p* < 0.001). Other characteristics, including marital status, smoking, alcohol use, and presence of chronic conditions, also varied significantly among groups, suggesting that distinct depression trajectories are associated with differentiated sociodemographic and behavioral profiles.

### 3.2 Longitudinal association between depressive symptom trajectories and diabetes onset risk

#### 3.2.1 Total depression score trajectory and diabetes onset risk

**Figure 2:**
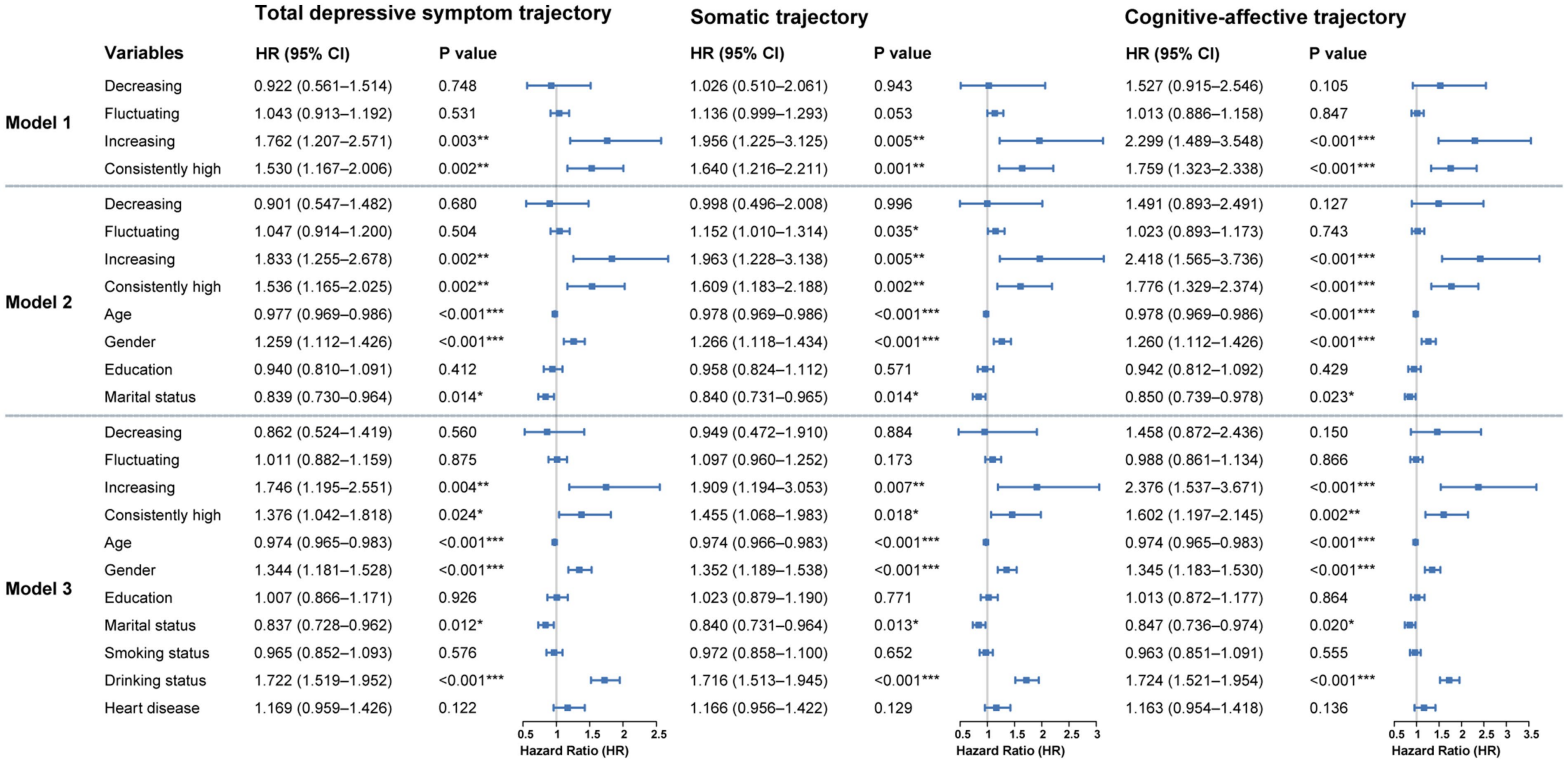
Cox regression results for the association between depressive symptom trajectories and diabetes onset over 10-year follow-up. Hazard ratios represent the relative risk compared to these reference categories. Model 1 is unadjusted. Model 2 is adjusted for demographic characteristics. Model 3 is further adjusted for health behaviors and chronic diseases. *p < 0.05, **p < 0.01, ***p < 0.001.

Using the consistently low group as the reference, results from the fully adjusted Cox proportional hazards model (Model 3) indicated a significantly increased risk of diabetes onset among individuals with an upward trajectory of total depressive symptom scores (HR = 1.746, 95%CI: 1.195–2.551, *p* = 0.004). A comparable elevation in risk was also identified in the consistently high group (HR = 1.376, 95%CI: 1.042–1.818, *p* = 0.024). In contrast, neither the decreasing nor the fluctuating trajectories showed statistically significant associations with diabetes risk compared with the consistently low group. The overall pattern was consistent in the unadjusted (Model 1) and partially adjusted (Model 2) models (Figure 2).

#### 3.2.2 Somatic symptom trajectories and diabetic onset risk

Model 3 demonstrated a significantly greater diabetes risk among individuals in the increasing group (HR = 1.909, 95%CI: 1.194–3.053, *p* = 0.007) and the consistently high group (HR = 1.455, 95%CI: 1.068–1.983, *p* = 0.018). No significant associations with diabetes risk were observed for the decreasing or fluctuating trajectories compared with the reference group. These associations were largely consistent across Model 1 and Model 2 (Figure 2).

#### 3.2.3 Cognitive-affective symptom trajectories and diabetic onset risk

In the cognitive-affective symptom trajectory analysis, model 3 showed a markedly higher risk of diabetes in the increasing group (HR = 2.376, 95% CI: 1.537–3.671, *p* < 0.001) and in the consistently high group (HR = 1.602, 95% CI: 1.197–2.145, *p* = 0.002). No significant associations were observed for the decreasing or fluctuating groups relative to the reference. Results from the unadjusted model as well as those partially adjusted for covariates (model 1 and model 2) exhibited consistent patterns (Figure 2).

### 3.3 Gender stratification

**Figure 3:**
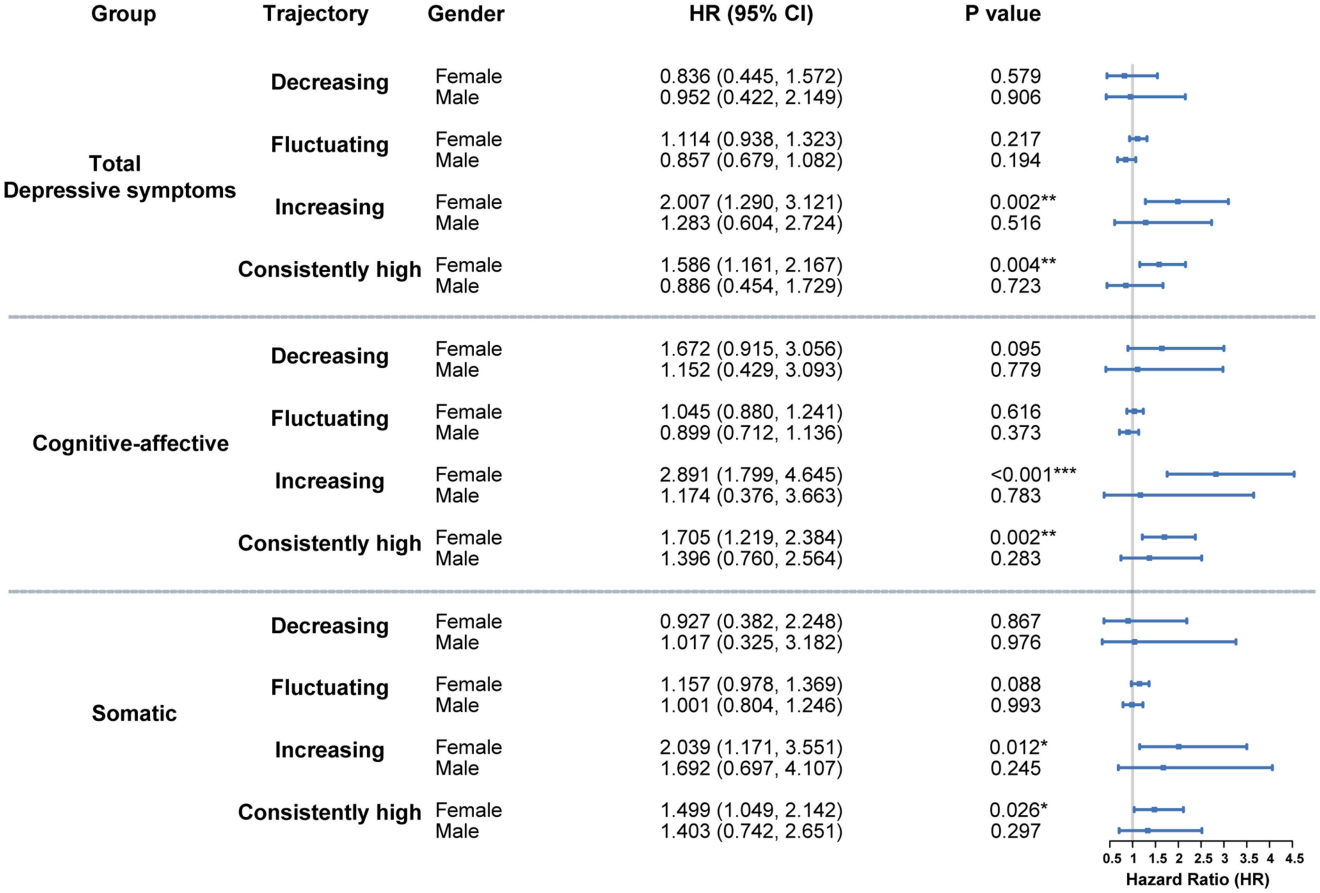
Gender-stratified cox analysis of the association between depressive symptom trajectories and diabetes risk. Hazard ratios represent the relative risk compared to these reference categories. Models were fully adjusted for demographic variables, health-related behaviors, and baseline chronic conditions. *p < 0.05, **p < 0.01, ***p < 0.001.

Gender served as a significant moderator in the association between depressive symptom trajectories and subsequent diabetes risk. As shown in Figure 3, no statistically reliable association was observed among males across any of the three trajectory types (total depressive symptoms, cognitive-affective, and somatic). Hazard ratios for all trajectory groups relative to the consistently low reference group were close to 1, with wide confidence intervals, suggesting minimal or unstable effects.

In contrast, females with either increasing or consistently high trajectories demonstrated strong associations with elevated diabetes risk across all symptom dimensions (Figure 3). For the total depressive symptom trajectory, HRs for the increasing and consistently high groups were 2.007 (95% CI: 1.290–3.121, *p* = 0.002) and 1.586 (95% CI: 1.161–2.167, *p* = 0.004), respectively. Within the cognitive-affective domain, HRs reached 2.891 (95% CI: 1.799–4.645, *p* < 0.001) for the increasing trajectory and 1.705 (95% CI: 1.219–2.384, *p* = 0.002) for the consistently high group. In the somatic domain, corresponding HRs were 2.039 (95% CI: 1.171–3.551, *p* = 0.012) and 1.499 (95% CI: 1.049–2.142, *p* = 0.026).

The findings indicate that persistent or worsening depressive symptoms in women—particularly within cognitive-affective and somatic domains—substantially heighten the probability of diabetes development. In contrast, no comparable associations were identified in men, suggesting sex-specific psychobiological mechanisms underlying metabolic outcomes.

## 4 Discussion

Using multi-wave follow-up data from two large longitudinal cohorts (HRS and ELSA), the analysis demonstrated a significant association between depressive symptom trajectories in middle-aged and older adults and subsequent risk of diabetes onset. Persistently high or progressively increasing patterns of depressive symptoms were linked to greater diabetes risk, with the cognitive-affective and somatic dimensions contributing most consistently to this association. Gender acted as a significant moderator; the relationship was evident among females, whereas no consistent or statistically significant variation in risk was observed among males.

This study characterized the somatic and cognitive-affective dimensions of depressive symptoms and identified their independent associations with diabetes risk. Somatic symptoms—such as reduced energy, sleep disturbances, and difficulty walking—more directly reflect disruptions in physiological functioning indicative of sustained chronic stress (24). Such stress can induce autonomic imbalance, diminish metabolic efficiency, and initiate chronic inflammatory responses, thereby substantially increasing insulin resistance and subsequent diabetes susceptibility (25, 26). In contrast, cognitive-affective symptoms—such as sadness, loneliness, and helplessness—are more closely associated with persistent negative emotional states that chronically activate the hypothalamic–pituitary–adrenal (HPA) axis. Prolonged HPA axis activation elevates circulating stress hormones (e.g., cortisol), sustains systemic inflammation, and progressively impairs pancreatic islet function and glucose regulation (27–29). Although their psychophysiological mechanisms differ, both symptom domains converge on common pathways involving chronic stress responses and metabolic dysregulation. Clinically, domain-specific integrative interventions are warranted: individuals with somatic symptoms may benefit from targeted improvements in sleep quality and restoration of physical vitality, whereas those with cognitive-affective symptoms may require psychotherapeutic support and cognitive-behavioral strategies. Such tailored approaches may achieve greater effectiveness in reducing long-term diabetes risk in middle-aged and older populations (30, 31).

Building on this, the results indicate that dynamic monitoring of depressive symptom trajectories may serve as a valuable adjunct in diabetes risk assessment. Identification of individuals with persistently high or progressively worsening depressive patterns enables earlier recognition of high-risk populations and supports more targeted screening and intervention strategies. Incorporation of both pharmacological and non-pharmacological treatment data in future research could further refine individualized management approaches and strengthen the evidence base for precision prevention and personalized medicine. Integrating longitudinal psychological assessment with metabolic monitoring may ultimately offer new opportunities for precision prevention, supporting the development of more effective, patient-centered strategies that consider both mental health trajectories and metabolic vulnerabilities.

The impact of persistent or progressively worsening depressive symptoms on diabetes risk may reflect the cumulative physiological consequences of prolonged exposure to chronic psychological stress (32). In contrast to transient emotional fluctuations, sustained increases or consistently elevated depressive symptom trajectories indicate reduced emotional resilience and the gradual accumulation of psychological vulnerability. This persistent strain affects neuroendocrine, behavioral, and metabolic systems, ultimately disrupting glucose homeostasis (33). Variations in trajectory patterns not only illuminate the course of symptom progression but also reveal underlying manifestations of chronic stress, emphasizing the value of incorporating a temporal dimension into intervention strategies. Depressive states are further associated with adverse health behaviors, including sedentary activity, irregular dietary habits, and substance use (e.g., smoking, alcohol consumption), which heighten the risk of impaired glycemic regulation (34). In addition, reduced adherence to treatment regimens and inadequate self-management in individuals with depression enhance metabolic susceptibility, reinforcing a cycle that elevates diabetes risk (35). Overall, neuroendocrine dysregulation, immune-inflammatory activation, and maladaptive health behaviors constitute interconnected pathways through which depression contributes to the development of diabetes.

The study identified a significant moderating effect of gender on the association between depressive symptom trajectories and diabetes risk. Depressive symptoms were linked to a marked increase in diabetes incidence among women, whereas no statistically significant association was observed in men. This gender-specific difference may be attributed to variations in biological responses to chronic psychological stress, including neuroendocrine regulation, hormonal fluctuations, and immune activity. Women appear more vulnerable to neuroendocrine disturbances—such as estrogen variability and heightened glucocorticoid secretion—under sustained stress, which may intensify inflammatory responses and disrupt metabolic homeostasis (36, 37). In addition, diabetes-related metabolic dysregulation may impose a greater burden on women. Evidence shows that among individuals with diabetes, women with comorbid depression exhibit poorer glycemic control than men, suggesting increased insulin resistance or impaired glucose metabolism in depressive states (38). Depressed women are also more likely to engage in emotional eating and experience greater weight gain, indicating a higher likelihood of obesity driven by unhealthy dietary behaviors, thereby contributing to an elevated risk of diabetes onset (39).

Several limitations warrant consideration. First, the observational design precludes definitive causal inference. Despite extensive covariate adjustment across multiple models, residual confounding and reverse causality cannot be fully excluded. Second, reliance on self-reported diagnoses of both depression and diabetes introduces potential information bias. In addition, the lack of data on antidepressant or psychotropic medication use may contribute to misestimation of the association magnitude. Finally, the absence of biochemical markers and biomarker assessments restricts the ability to clarify underlying biological mechanisms.

## 5 Conclusion

The analysis demonstrates a significantly increased risk of incident diabetes among middle-aged and older individuals with either consistently high or rising depressive symptom trajectories, with stronger associations observed in women. Both cognitive-affective and somatic symptom trajectories independently predict diabetes onset, suggesting potentially distinct mechanistic pathways that connect specific dimensions of depressive symptoms with metabolic outcomes.

## Data Availability

The raw data supporting the conclusions of this article will be made available by the authors, without undue reservation.
